# Time changes in the spectrum of urinary stone composition: a role for climate variations?

**DOI:** 10.1186/s12882-020-02193-x

**Published:** 2020-12-09

**Authors:** Alberto Trinchieri, Alessandro Maletta, Giovanni Simonelli, Luca Boeri, Elisa De Lorenzis, Emanuele Montanari

**Affiliations:** 1 Department of Urology, IRCCS Ca’ Granda Ospedale Maggiore Policlinico, University of Milan, Via Commenda 15, 20100 Milan, Italy; 2 Department of Urology, Manzoni Hospital, Lecco, Italy

**Keywords:** Urinary calculi, Epidemiology, Climate, Calcium oxalate, Uric acid, Struvite

## Abstract

**Background:**

An increase of the frequency of uric acid urinary stones compared to calcium-containing ones has been recently described. This study was aimed at assessing the frequency of different types of urinary stones in the population of northern Italy in the period 2016–18 compared to 2001–2003.

**Methods:**

Analyses by infrared spectroscopy of 1007 stones endoscopically removed at two institutions in the area of Milan (Northern Italy) were retrospectively considered. Stones were classified as calcium oxalate monohydrate (COM) and dihydrate (COD), mixed uric acid/calcium oxalate (UC); uric acid (UA), struvite (ST); apatite (CAP); mixed calcium oxalate / apatite (CAPOX); others. The patients were divided into two groups: 2001–2003 and 2016–2018. The average temperature values of the region over the two time periods were obtained by the national statistical institute.

**Results:**

The average age of the 2001–2003 group (45.8+/− 15.4 years) was significantly lower than the average age of the 2016–18 group (57.9+/− 14.8) (0.000). M / F ratio was similar in the two groups: 119 / 69 (1,0.58) in 2001–2003 and 527 / 292 (1,0.55) in 2016–18 (*p* = 0.862). COM stones tended to more frequent in 2016–18 group than in 2001–03. COD stones were significantly more frequent in 2001–03 than in 2016–18. ST stone frequency was increased from 2001 to 03 to 2016–18. No increase of uric acid containing stones was observed in 2016–18. Results were confirmed after adjustment by age. Averages annual regional temperatures increased from 14 °C to 15.4 °C during the two observation periods.

**Conclusions:**

No increase of UA stones was observed, probably due to the limited impact of the global warming in our temperate climate.

## Background

Urinary stones may have different composition in relation to their aetiology, which results in different conditions of urinary saturation favoring the formation of stones of calcium oxalate, calcium phosphate, struvite, uric acid, sodium or ammonium urate, apatite, or, more rarely, cystine, 2,8-dihydroxyadenine and xanthine.

The frequency of different types of stones varies in different populations due to the variability of the environmental factors, such as diet and climate, and, for the same reason, can vary over time in the same population.

Recently, some authors observed in some Western countries a tendency to change the composition of urinary stones with an increase of the frequency of uric acid stones compared to calcium-containing ones [1, 2].

The formation of uric acid stones depends on genetic and environmental risk factors and may be associated with obesity and diabetes [3–5]. The role of diet in the pathogenesis of uric acid stone disease is not fully defined, although the formation of uric acid stone has been associated with increased protein and alcohol consumption [6]. On the other hand, there is evidence that uric acid stone formation is favored by high ambient temperatures which cause an increase in skin fluid losses resulting in a reduction in urinary volumes and urinary pH values, both promoting crystallization of uric acid. A narrative review of the estimated prevalence of uric acid nephrolithiasis around the world demonstrated that higher rates were observed in countries with hot and dry climates [7]. In a study comparing the composition of urinary stones from seven areas of the United States, a higher frequency of uric acid stones was observed in Florida, one of the states with the highest average temperature and humidity [8].Furthermore, a recent study showed that the risk of acutely emergent stone episodes after exposure to short periods of high wet-bulb temperatures was higher in men who form uric acid stones more frequently than in women [9]. Consequently, the effect of climate on the risk of uric acid stones could be related to relatively short heath events (“heath waves”) rather than to the increase in average temperature.

This study was aimed at assessing the frequency of different types of urinary stones in the population of northern Italy in the three-year period 2016–18 compared to 2001–2003 and analyzing potential environmental risk factors that may have affected the spectrum of stone composition.

## Methods

Analysis of stones endoscopically removed at two institutions that collaborate in clinical, educational and research activity were considered retrospectively. For the period 2001–2003 the results of the stone analyses from Milan were evaluated, while for the period 2016–2018 were considered stones both from Milan and Lecco, in the Lombardy region of Northern Italy. The stones were examined with infrared spectroscopy from the laboratory of Milan.

To allow a statistical evaluation of the results, the stones were arbitrarily grouped into some categories in accordance with what was previously reported by other authors. Eight main groups have been identified: 1) calcium oxalate monohydrate (COM) = calculi containing> 50% of calcium oxalate (in the absence of a minor component > 10% of uric acid or calcium phosphate); 2) calcium oxalate dihydrate (COD) = stones containing> 50% of calcium oxalate (in the absence of a minor component > 10% of uric acid or calcium phosphate); 3) mixed calculi of calcium oxalate and uric acid (> 10%)(UC); 4) anhydrous uric acid (without calcium oxalate component)(UA); 5) struvite (ST) = presence of at least 10% struvite; 6) calcium phosphate (CAP) = calcium phosphate> 50%); 7) calcium oxalate and phosphate mixed stones (in the presence of a 10–50% phosphate component (CAPCAOX; 8) uric acid dihydrate, sodium urate, ammonium urate, brushite and cystine stones were observed less frequently and were reported as other stones (OTHERS). Age and sex of the patients were obtained from the patient records.

The patients were divided into two groups, one for patients observed during the 2001–2003 period and the other for patients observed in the 2016–2018 period.

The average temperature values of the region over the two time periods were obtained by the national statistical institute (ISTAT) [10].

### Statistics

Age of patients was expressed as mean ± standard deviation. Differences between groups were assessed by Student’s independent t-test or one-way ANOVA with post hoc multiple comparisons according to Bonferroni. Frequencies of the different types of stones in the groups were reported as contingency tables and chi square was used to test the significance of associations.

To avoid possible bias related to the different age distribution of patients in the different groups that could affect the crude rate of some types of stones that are more frequent in specific age groups, age-standardized rates of the frequency of different stone types were calculated in the populations studied.

Age adjustment was made by considering the distribution in age classes of a standard population constructed by combining the three populations under evaluation (Table 1).

**Table 1:** Distribution of cases by age groups

Age Group	2001–03 MI	2016–18 LC	2016–18 MI	2016–18 LC + MI	Age distribution Standard Population
< 30	31 (16.4%)	17 (3.7%)	20 (5.7%)	37 (4.5%)	68 (7%)
31–40	46 (24.4%)	34 (7.4%)	26 (7.5%)	60 (7.3%)	106 (11%)
41–50	42 (22.3%)	77 (16.8%)	72 (19.8%)	149 (18.2%)	191 (19%)
51–60	30 (15.9%)	111 (24.3%)	99 (26.5%)	210 (25.6%)	240 (24%)
61–70	28 (14.8%)	93 (20.4%)	76 (21.3%)	169 (20.6%)	195 (19%)
> 70	11 (5.8%)	124 (27.1%)	70 (18.9%)	194 (23.7%)	205 (20%)
Total	188	456	363	819	1007

2001–2003 MI = observed in Milan period 2001–03

2016–2018 LC = observed in Lecco period 2016–18

2016–2018 MI = observed in Milan period 2016–18

2016–2018 LC + MI = observed in Lecco and Milan 2016–18

The Statistical Package for the Social Sciences version 11.5 for Windows was used for statistical analysis. These comparisons were considered as significantly different if *P* < 0.05.

## Results

The average age of the 2001–2003 group (45.8+/− 15.4 years) was significantly lower than the average age of the 2016–18 group (57.9+/− 14.8) (0.000).

The distribution by age group is shown in Table 1 (*p* = 0.000).

M / F ratio was similar in the two groups: 119 / 69 (1:0.58) in 2001–2003 and 527 / 292 (1:0.55 in 2016–18 (*p* = 0.862).

The results of 1007 stone analyses have been evaluated. Stones were classified as COM in 500, COD in 112, UC in 54, UA in 131, ST in 46; CAP in 57 and CAPCAOX in 87. Other less frequent types of calculus have been observed in 20 patients (ammonium urate, sodium urate, uric acid dihydrate, brushite, cystine).

The distribution by stone type in the 2016–18 and in 2001–03 groups are reported in Table 2.

**Table 2:** Frequency of different type of stones in the 2001–03 and 2016–18 groups

	COM	COD	UC	UA	ST	CAP	CAPCAOX	Others	Total
2001–03 MI	82 (44%)	33 (18%)	6 (3%)	28 (15%)	3 (1%)	13 (7%)	16 (9%)	7 (3%)	188
2016–18 LC + MI	418 (51%)	79 (10%)	48 (6%)	103 (13%)	43 (5%)	44 (5%)	71 (9%)	13 (2%)	819

*COM* calcium oxalate monohydrate; *COD* calcium oxalxte dihydrate; *UC* mixed uric acid/calcium oxalate; *UA* uric caid; *ST* struvite; *CAP* apatite; *CAPCAOX* mixed apatite/calcium oxalate

COM stones tended to be more frequent in 2016–18 group than in 2001–03 group (51.0% vs 43.6%) but difference was not significant (*P* = 0.066). On the contrary, COD stones were less frequent in 2016–18 group than in 2001–03 group (9.6% vs 17.5%, *P* = 0.002). The frequency of uric acid containing stones (18.4% vs 18.0%, *P* = 0.917) and of calcium phosphate stone (CAP+CAPCAOX) (14% vs 15.4%, *P* = 0.625) was not different between 2016 and 18 and 2001–03. Struvite stones frequency was higher in 2016–18 than in 2001–03 (5.2% vs 1.6%, *P* = 0.035). Trends after age-adjustment confirmed the differences of crude rates although difference for COM age-adjusted rates between 2016 and 18 became stronger (54.1% vs 43.7%) and uric acid-containing stones tended to be even less frequent in 2016–18 (16.9% vs 24.7%). After age-adjustment COD stones were less frequent in 2016–18 (9.1% vs 13.7%), struvite stones more frequent in 2016–18 (5.5% vs 1.1%) and no difference was observed for calcium phosphate stone frequency (CAP+CAPCAOX) (14.5% vs 13.3%). Mean age and M/F ratio for different types of stones are shown in Table 3.

**Table 3:** Mean age and M/F ratio for different types of stones

	COM	COD	UC	UA	ST	CAP	CAPCAOX	Others	Sig
N°	500	112	54	131	46	57	87	20	
Mean age (years)	53.7+/−15.9	53.7+/−15.9	58.5+/−16.7	66.4+/−11.5	56.7+/−17.9	50.1+/−14.7	50.4+/−13.4	46.1+/−18.1	0.000
M/F	330/170 (1:0.51)	79/33 (1:0.42)	36/18 (1:0.50)	109/22 (1:0.20)	21/25 (1:1.19)	26/31 (1:1.19)	41/46 (1:1.12)	9/11 (1:1.22)	0.000

Post hoc analysis of difference of age according to Bonferroni

UA > COM = 0.000; UA > COD = 0.000; UA > ST = 0.005; UA > CAP = 0.000; UA > CAPCAOX = 0.000; UA > others = 0.000

UC > CAPCAOX = 0.028; UC > others = 0.031

*COM* calcium oxalate monohydrate; *COD* calcium oxalxte dihydrate; *UC* mixed uric acid/calcium oxalate; *UA* uric caid; *ST* struvite; *CAP* apatite; *CAPCAOX* mixed apatite/calcium oxalate

## Discussion

The prevalence of urinary calculi and the frequency of different types of stone can change consequently to changes of environmental factors, such as diet and climate, as well as changes of age and gender distribution of the population.

Our series confirm some findings commonly observed in epidemiological studies of urolithiasis such as higher struvite and apatite-containing stone rates in females and higher rates of uric acid containing stones in male and older patients.

Only a limited number of studies have assessed the spectrum of the composition of urinary calculi in the same population over different periods of time [1, 2, 11–15].

In South Australia urinary stone composition has remained relatively static over the past 30 years [11].

In Japan, from 1953 to 1984, a reduction in the frequency of struvite calculi from 20% before 1960 to 10% after 1961 was observed, although a slight tendency to increase was again detected after 1973, especially in females [12]. In Massachusetts from 1990 to 2010, the frequency of struvite stones significantly decreased from 7.8 to 3.0% in females but remained stable in males ranging from 2.8 to 3.7% [13],

This trend is in accordance with our observations that show a decrease from 24% of our previously reported observations in the period 1981–95 [16] to the actual 1.6–6.5% value. The frequency of struvite stones was very low in the 2001–03 series in Milan (1.6%), although it is interesting to note that in the most recent period it tended to increase slightly from 3.3 to 6.5%. This can be explained by recent immigration in Italy from countries of Eastern Europe and from north and central Africa, which contributed with a relevant number of infection stone cases due to poor health conditions of those countries. In Canada, an 80.4% rate of calcium oxalate and/or calcium phosphate stones was observed with a 3.9% rate of magnesium ammonium phosphate and 7.6% of uric acid/urate stones [14]. In the calcium stone group, stones with prevalent oxalate content accounted for 65% and stones with prevalent phosphate content for 16%, although from 1980 to 83 to 1995–1998 a relative increase in stones with prevalent oxalate and a decrease in stones with prevalent phosphate was observed. A similar trend was reported in Massachusetts with a significant decrease from 20 to 11.7% of the frequency of apatite stones in females but an increase from 9.8 to 12.5% in males [13]. In our series we observed only a slight decrease of the frequency of apatite-containing stones from 15.4 to 14%, in association with an increase of the rate of COM and a decrease of COD stones.

In Texas, uric acid containing stones increased from 7 to 14% from 1980 to 2015 [1]. This trend was explained by an increase in age and body mass index (BMI) in the population of stone formers. However, in Minnesota [15], from 1984 to 2012, no change of uric acid stone rate was observed and, in Massachusetts, from 1990 to 2010, the rate of uric acid stones increased in females, from 7.6 to 10.8%, but not in males (11.7 vs 10.8%) [12].

In southern Italy it was also observed from the period 1983–86 to 2008–2011, a decrease in the frequency of calcium oxalate stones (from 83.9 to 76.6%) and an increase in the frequency of uric acid stones from 2.2 to 9.3% [2].

On the contrary, the results of our study do not confirm this trend, demonstrating unchanged rates of uric acid stone rates over the last 15 years even after age adjustment.

The increase of the frequency of uric acid stones in some countries could be related to different factors. The main driving force of uric acid crystallization in urine is an acidic pH that can be associated with ageing, obesity and type 2 diabetes. Ageing is a common feature of all Western populations. According to the World Population Prospects 2019 of the United Nations [17] the ratio of population aged > 65 years (old-age dependency ratio) increased in the United States from 20.9% in 2000 to 24.6% in 2015 and in Italy from 29.5 to 36.8%. Obesity is the result of chronic imbalance of energy intake and expenditure by physical activity and unhealthy diet and a sedentary lifestyle are also considered important drivers of the onset of diabetes in genetically predisposed subjects. According to NCD Risk Factor Collaboration [18], obesity prevalence (BMI > 30 kg/m2) in the United States increased from 25.5% in 2001 to 36.4% in 2016 for men and from 28.5 to 38.1% for women. Obesity rates in Italy were lower although in the same time interval they increased from 14.9 to 20.9% in men and from 16.8 to 20.4% in women. Crude prevalence of diabetes in the United States increased from 7.7% in 2001 to 9.8% in 2014 in men and from 6.9 to 8.3% in women. In Italy, a similar increase was observed from 7.5 to 9.5% of men and from 6.9 to 7.4%, although these values refer to an older population [19].

All these factors should lead to an increase in the frequency of uric acid stones which has not been uniformly observed in all the studies we have evaluated.

We must therefore assume that there is another epidemiological factor which is decisive in increasing the frequency of uric acid stones in certain geographical regions.

**Fig. 1:**
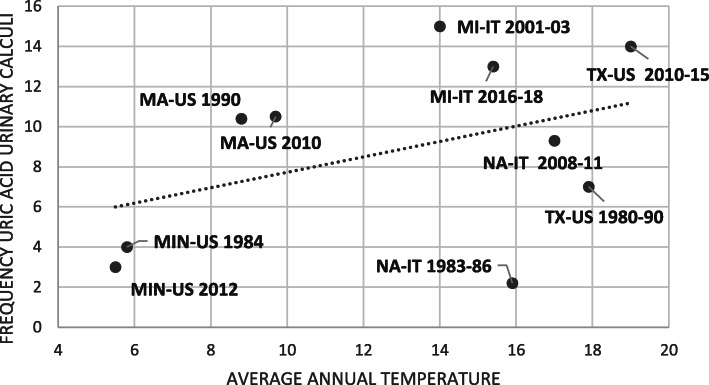
Average temperature values from official weather websites plotted against the frequency values of uric acid calculi in different series (Massachusetts = MA, Minnesota = MIN, Texas = TX, Milan = MI, Naples = NA)

Different climatic conditions could be an explanation of the different trends of the epidemiology of uric acid urinary calculi observed in different studies (Fig. 1).

Uric acid stones are more easily formed in a warm climate. In South Australia a greater incidence of uric stones during Summer and Autumn was observed [20] and Stuart et al. showed that in the warmer months of the year there is a higher level of urinary saturation for uric acid in relation to lower urinary volumes and lower urine pH values [21].

In fact, as a result of global warming, the average annual temperatures increased by about one Celsius degree from the 1981–85 period to the 2011–2015 period in most of United States [22].

According to data collected by the National Climatic Data Center of the United States [22], average annual temperature increased from 5.2–6.4 °C to 3.7–7.3 °C in Minnesota, from 8.4–9.2 °C to 8.7–10.7 °C in Massachusetts and from 17.4–18.3 C° to 18.2–19.9 °C in Texas.

In Texas, the increase of temperature may have had a greater impact on the risk of formation of uric acid stones as it occurred in a higher temperature range than in northern regions of the United States (Fig. 1).

In fact, the number of days in which the risk threshold for crystallization of uric acid was exceeded was higher. The threshold value beyond which the risk of precipitation of uric acid in the urine is significantly increased has not been precisely defined, but it can be inferred by values of temperature associated with the risk of the onset of acute gout attacks, that according to Neogi et al. could be placed at 26 °C [23].

Similarly, the different climatic conditions in southern Italy may explain the discrepancy of the study of Rendina et al. [2] with the results of our study carried out in a region of northern Italy [3].

A possible bias factor in our study is the imbalance between the number of patients retrospectively reviewed in the two study periods in favor of patients observed in the most recent period. Unfortunately, in the first period the use of infrared spectrophotometry had not been routinely extended to all patients, whereas in the most recent period the analysis was carried out routinely. In addition, the volume of patients observed in the second period increased because a second institution was associated with the primary academic institution. Consequently, the populations studied were homogeneous and the increase in numbers of the second group is not attributable to an increase in the prevalence of urinary stones which, after the increase observed in the 1980s, [24] has remained relatively stable in Italy over the last decade at a value of 7.5% [25].

## Conclusions

In conclusion, in the northern regions of our country we have not observed an increase in uric acid stones, as described in other countries, probably as a result of the limited impact of global warming in a region characterized by high mountains and great lakes.

## Data Availability

The datasets during and/or analyzed during the current study available from the corresponding author on reasonable request.

## Abbreviations

COM: Calcium oxalate monohydrate
COD: Calcium oxalate dihydrate
UC: Mixed uric acid/calcium oxalate
UA: Uric acid
ST: Struvite
CAP: Apatite
CAPOX: Mixed calcium oxalate / apatite
M / F: Male to female
BMI: Body mass index
